# A comparative analysis of sequence composition in different lots of a phage display peptide library during amplification

**DOI:** 10.1186/s12985-024-02600-x

**Published:** 2025-02-01

**Authors:** Anders Wilgaard Sinkjaer, Ane Beth Sloth, Amanda Oester Andersen, Malte Jensen, Babak Bakhshinejad, Andreas Kjaer

**Affiliations:** 1 Department of Clinical Physiology and Nuclear Medicine, Copenhagen University Hospital-Rigshospitalet, Blegdamsvej 9, 2100 Copenhagen, Denmark; 2 Cluster for Molecular Imaging, Department of Biomedical Sciences, Copenhagen University Hospital-Rigshospitalet, University of Copenhagen, Blegdamsvej 3B, 2200 Copenhagen, Denmark; 3 Department of Otorhinolaryngology, Head & Neck Surgery and Audiology, Copenhagen University Hospital-Rigshospitalet, Blegdamsvej 9, 2100 Copenhagen, Denmark

**Keywords:** Amplification, Bias, Compositional heterogeneity, Enrichment factor, Illumina sequencing, Motif discovery, NGS, Peptide library, Phage display, Propagation-related peptides

## Abstract

**Background:**

To develop efficient selection strategies and improve the discovery of promising ligands, it is highly desirable to analyze the sequence composition of naïve phage display libraries and monitor the evolution of their peptide content during successive rounds of amplification. In the current study, we performed a comparative analysis of the compositional features in different lots of the same naïve phage display library and monitored alterations in their peptide compositions during three rounds of amplification.

**Methods:**

We conducted three rounds of duplicate serial amplification of two different lots of the Ph.D.™-12 phage display library. DNA from the samples was subjected to Next-Generation Sequencing (NGS) using an Illumina platform. The NGS datasets underwent a variety of bioinformatic analyses using Python and MATLAB scripts.

**Results:**

We observed substantial heterogeneity in the sequence composition of the two lots indicated by differences in the enhanced percentage of wildtype clones, reduced diversity (number of unique sequences), and increased enrichment factors (EFs) during amplification as well as by observing no common sequence between lots and decreased number of common sequences between the naïve library and the consecutive rounds of amplification for each lot. We also found potential propagation-related target-unrelated peptides (TUPs) with the highest EFs in the two lots, which were displayed by the fastest-propagating phage clones. Furthermore, motif analysis of the most enriched subpopulation of amplified libraries led to the identification of some motifs hypothesized to contribute to the increased amplification rates of the respective phage clones.

**Conclusion:**

Our results highlight tremendous heterogeneity in the peptide composition of different lots of the same type of naïve phage display library, and the divergent evolution of their compositional features during amplification rounds at the amino acid, peptide, and motif levels. Our findings can be instrumental for phage display researchers by bringing fundamental insights into the vast extent of non-uniformity between phage display libraries and by providing a clear picture of how these discrepancies can lead to different evolutionary fates for the peptide composition of phage pools, which can have profound impacts on the outcome of phage display selections through biopanning.

**Supplementary Information:**

The online version contains supplementary material available at 10.1186/s12985-024-02600-x.

## Introduction

Since the invention of phage display by George Smith [1], this technique has been used for the discovery of many peptides with potential applications in a variety of biomedical areas [2]. Phage display is an *in vitro* evolution methodology based on the physical connection between genotype (the nucleic acid sequence encoding a ligand) and phenotype (the expressed ligand on the phage surface), allowing for the identification of amino acid sequences that bind specifically to a variety of targets. The introduction of peptide libraries has developed phage display into a high-throughput approach by selection of hugely diverse combinatorial libraries (with millions of different peptide sequences) towards a given target. Peptide phage display has been used for the isolation of high-affinity binders to numerous relevant targets [2, 3], which represent a remarkable potential for the development of targeted gene and drug delivery [4–7] and imaging platforms [8–10].

The screening of phage display peptide libraries is conducted through the procedure of biopanning. This consists of two steps: a selection step in which phages bearing peptides with high binding affinity to the target are enriched and an amplification step in which already enriched phage clones are increased in copy number to yield a new pool as input for the subsequent round of biopanning [11]. Amplification is performed by infecting the host bacterium with the enriched phage clones, typically a laboratory strain of *E.coli*, followed by precipitation of phage particles through extraction. Biopanning is an iterative process that is repeated in multiple rounds to obtain binders of sufficiently high affinity to the target [12]. Library selections through biopanning have shown promise in the identification of many target-specific ligands [13–17].

In recent years, next-generation sequencing (NGS) has been coupled to phage display, allowing the sequencing of millions of clones in parallel. There is an increasing body of research using NGS for analysis of the results of biopanning outputs [18–20]. However, there is a limited number of studies using NGS for investigation of the naïve unselected phage display peptide libraries [21–24]. These studies have revealed compositional bias in the naïve phage display libraries. Amplification, which is an integral component of biopanning, is known as one of the major causes of the inception of bias in phage display libraries [25–27]. Amplification leads to the unwanted enrichment and isolation of propagation-related target-unrelated peptides (Pr-TUPs). These are peptides that are displayed on phages with a higher propagation rate, leading them to become enriched in the pool in a target-independent manner. Thus far, phage display research has witnessed the use of some experimental [28, 29] and bioinformatic approaches [30] for Pr-TUP identification. It has already been demonstrated that a significant number of Pr-TUPs might exist in the phage display peptide libraries [31]. Due to their nonspecific enrichment during phage display selection, Pr-TUPs hold considerable potential for misinterpretation of the data obtained from biopanning. If these peptides remain unidentified, they can be mistaken as target-related peptides, posing an enormous challenge for the translation of peptides identified by phage display into the clinic. Therefore, it is highly important to clean biopanning data by identifying Pr-TUPs and removing them from the pool of selected peptides [27]. There are few studies on utilizing NGS for analysis of amplified phage display libraries and investigation of the evolution of phage pools during amplification [31–33], and to our knowledge, there is no report based on a comparative study of compositional features in different lots of a naïve phage display library and how they evolve differently during amplification.

In the current work, we used NGS to perform a comparative analysis on two lots of the Ph.D.™-12 Phage Display Peptide Library and monitor alterations occurring in the library during successive amplification rounds. The Ph.D.™-12 Phage Display Peptide Library (manufactured by New England Biolabs) is the most widely used phage display peptide library. Our results highlight tremendous heterogeneity in the sequence composition of different lots of the same type of phage display library, leading to divergent evolutionary trajectories during amplification for these lots. Additionally, we identified and reported propagation-related peptides, which are displayed on fast-propagating phage clones, enriched during serial amplification of the two lots. We also conducted an extensive motif analysis on the naïve and amplified phage pools of the two lots and found motifs hypothesized to contribute to the higher amplification rate of phage clones in the library.

## Results

### Lots of the library show differential changes in the frequency of wildtype and unique clones during amplification

**Fig. 1:**
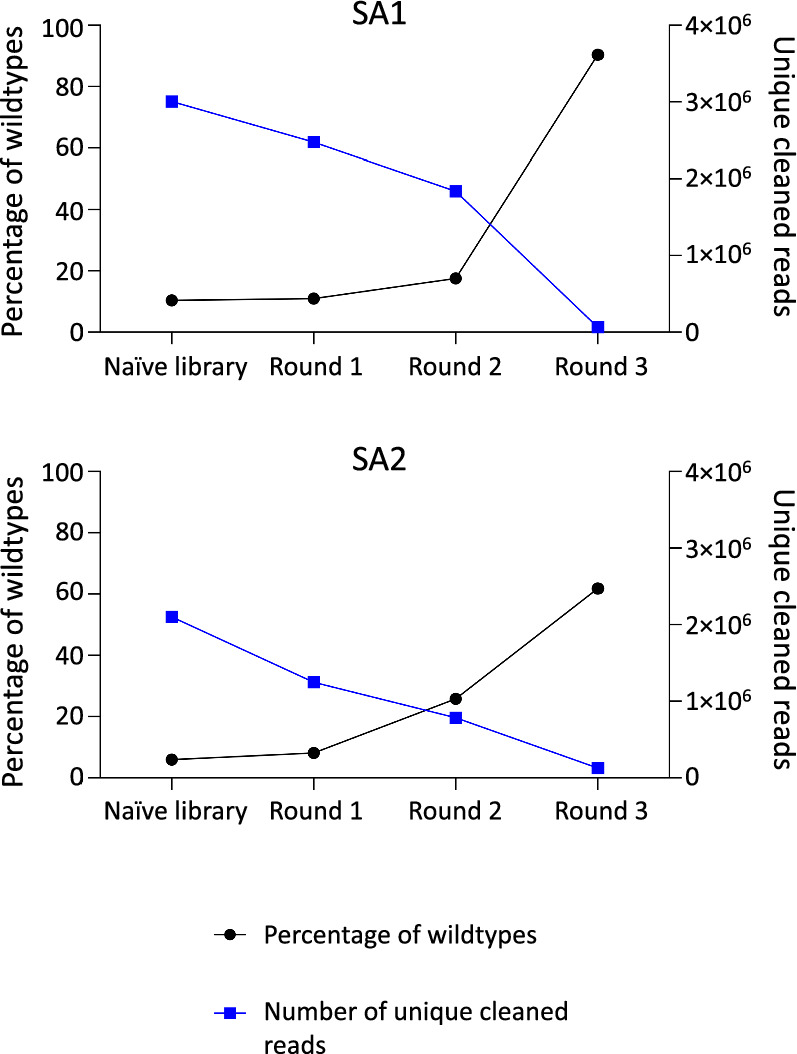
Overview of the percentage of wildtypes (black) and the number of unique reads (blue) in the two lots in the naive library and through the different rounds of amplification. SA1: Serial amplification experiment 1 and SA2: Serial amplification experiment 2

The FASTQ files from NGS were processed using Python scripts. The files were cleaned based on their Phred score and whether they contained a peptide insert or not (See Materials and Methods; Sect. Analysis of NGS data). For each sample, the total number of reads, the number of cleaned reads, and the number of removed reads are found in Supplementary Tables A1 and A2. Additionally, the percentage of reads that did not contain a peptide insert was determined. These were denoted as the percentage of wildtype reads (Supplementary Tables A1 and A2). It was found that the frequency of wildtypes increases during rounds of amplification in both lots. The naïve library used in SA1 contains 10.36% wildtypes and during the experiment, this increases to an average of 90.37% in round 3. In the naïve library used in SA2, the percentage of wildtypes is lower (5.99%), and the amplified phage pool also represents a lower percentage of wildtypes in round 3 (an average of 61.81%). The number of unique reads shows variation in the naïve library of two lots and is decreased during amplification rounds with the largest reduction in round 3. These findings are illustrated in Fig. 1. Although the increased frequency of wildtype clones and reduced number of unique clones are observed in both SA1 and SA2, the two different lots of the library display a remarkable discrepancy in quantitative changes of these compositional features both in the naïve library and during serial amplification-driven evolution of the library.

**Fig. 2:**
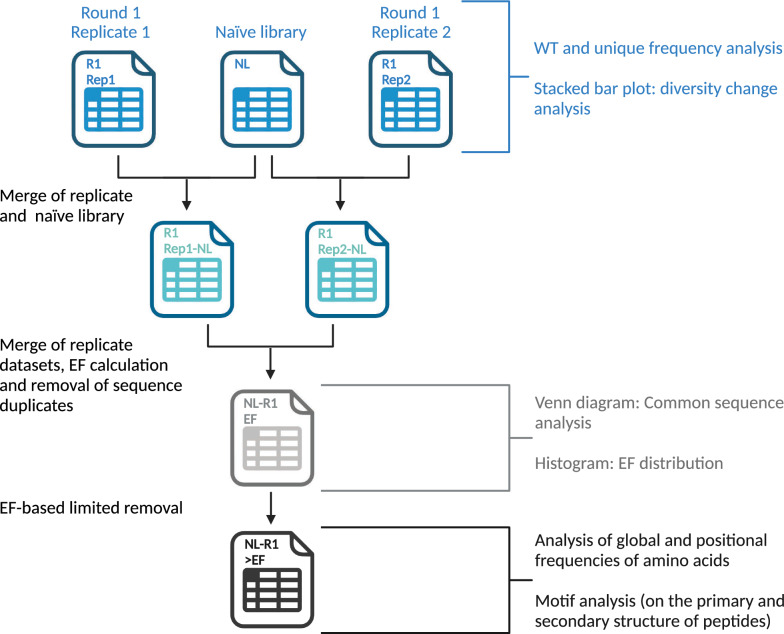
Overview of how the merged files representing sequences from different replicates were generated and how the generated files were used for different analyses. The figure was created with BioRender.com

For subsequent analyses, the replicates in each round were merged. The merged files also contained the calculation of EF. The merged files containing all sequences were used to prepare the stacked bar plots (Sect. Diversity changes follow lot-specific patterns during amplification). All sequences with an EF value were used for the analysis of overlapping sequences between amplification rounds (Sect. The library lots are completely heterogeneous in sequence composition and become more homogeneous during amplification) and the EF distribution (Sect. The distribution of enrichment factors of peptides becomes more divergent during amplification with substantial discrepancies between lots). For the analysis of global and positional frequencies of amino acids (Sect. Global and positional frequencies of amino acids indicate distinct compositional changes between lots and during amplification), the top 1000 sequences with EF > 1 were used. For motif analysis (Sect. Motif analysis led to the identification of some motifs with probable association with a higher amplification rate of phage clones), the top 18,622 sequences with EF > 1 were included for motif discovery in the primary structure of peptides and all sequences with EF > 1 were used for motif discovery in the secondary structure of peptides. This is illustrated in Fig. 2.

### Diversity changes follow lot-specific patterns during amplification

**Fig. 3:**
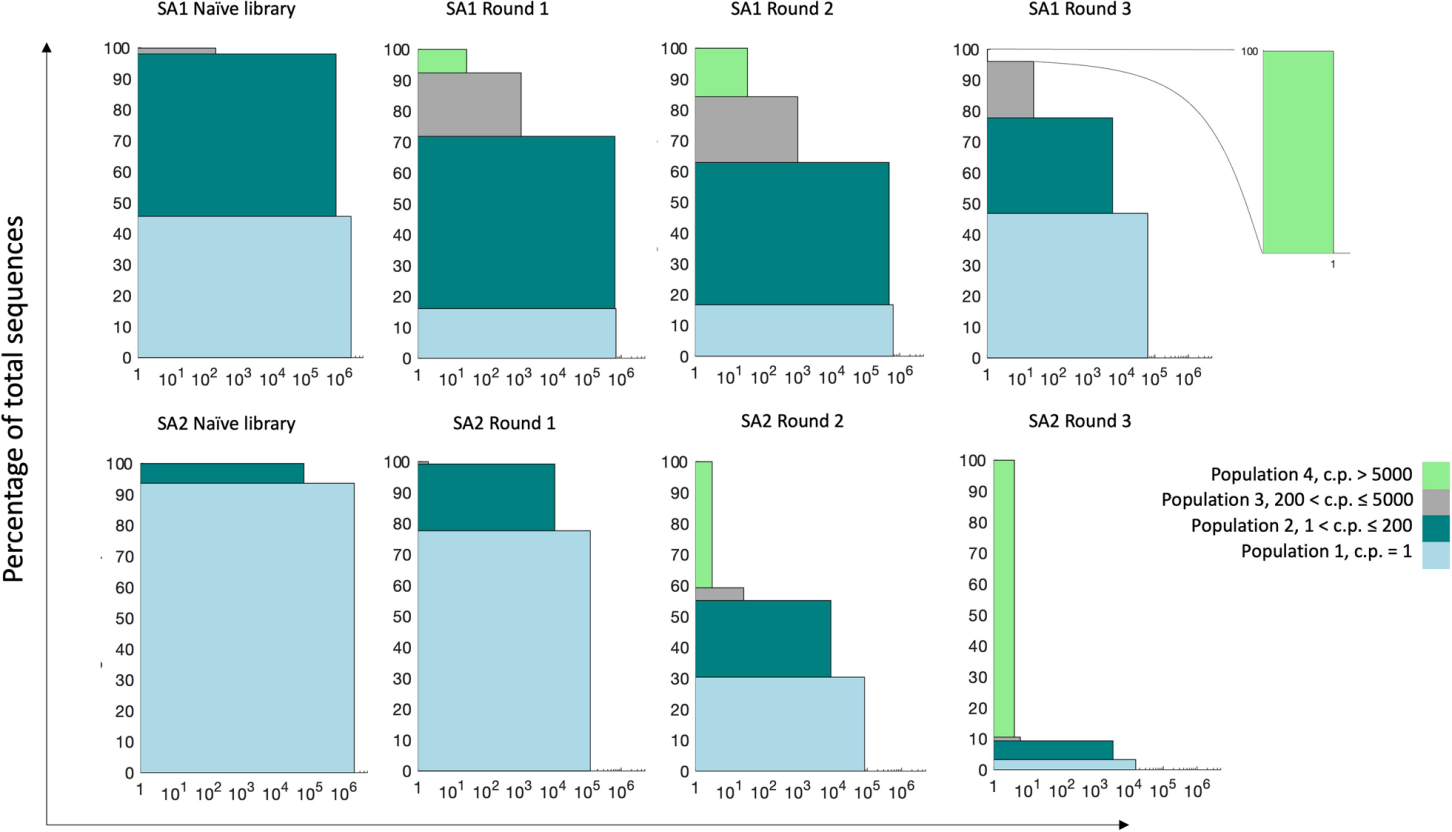
Stacked bar plots visualizing the frequency distribution of sequences observed in NGS data from the SA1 and SA2 experiments. Peptide sequences were grouped into bins (shown by different colors) according to their copy number. c.p.: copy number

To assess the diversity of peptide pools, the percentage of distinct sequences was calculated in the naive library and during amplification for each lot. The results can be found in Supplementary Tables A1 and A2 for SA1 and SA2, respectively. In the naïve library, considerable differences in the percentage of distinct sequences can be observed between lots. In the naïve library used in SA1 experiment, there are 61.89% distinct sequences, whereas the percentage of distinct sequences in the naïve library for SA2 experiment is 96.66%. The biggest alterations in the percentage of distinct sequences during amplification are observed in SA2 (ranging from 96.7% in the naïve library to an average of 24.6% after the third round of amplification). In SA1, the percentage of distinct sequences exhibited less variation during serial amplification (61.9% in the naïve library and an average of 57.7% after the third round of amplification). We also investigated the composition of different lots, both the naïve and amplified libraries, by cataloging peptides into frequency-based populations and illustrating the distribution of different populations within each sample through stacked bar plots. Here, each sample was grouped into bins of different frequencies (Fig. 3). Through rounds of amplification, it is expected that the population of singletons (sequences with a single copy) decreases, and peptide sequences with high abundances increase in copy number.

There was a remarkable difference in the proportion of singletons between the naïve libraries of the two lots (45.58% for SA1 and 93.62% for SA2). Furthermore, both lots indicate the trend of a decreasing singleton population as well as an increase in the population of repeated sequences (i.e., non-singletons) during amplification, which was reflected by enlargement of the previously existing bins representing non-singletons (with lower frequencies) and appearance of new bins representing highly frequent peptides. Of note, round 3 of SA1 was an exception, which exhibited an increased percentage of the singleton population. This observation might be ascribed to the higher diversity of the singleton population in SA1 compared to SA2 (Supplementary Table A3), leading to less elimination of the members of this population from the SA1 phage pool. However, the enhanced percentage of singletons in round 3 of SA1 was accompanied by a decrease in the number of unique sequences in the singletons, highlighting a decline in the size of this population similar to other rounds of amplification. The data used to generate Fig. 3 can be found in Supplementary Table A3. In general, the reduced percentage of distinct sequences as well as the decreased number of singletons provides evidence for the diminished diversity of phage display libraries during serial amplification of the pool and that there is a substantial heterogeneity between lots in the observed pattern of diversity changes.

### The library lots are completely heterogeneous in sequence composition and become more homogeneous during amplification

**Fig. 4:**
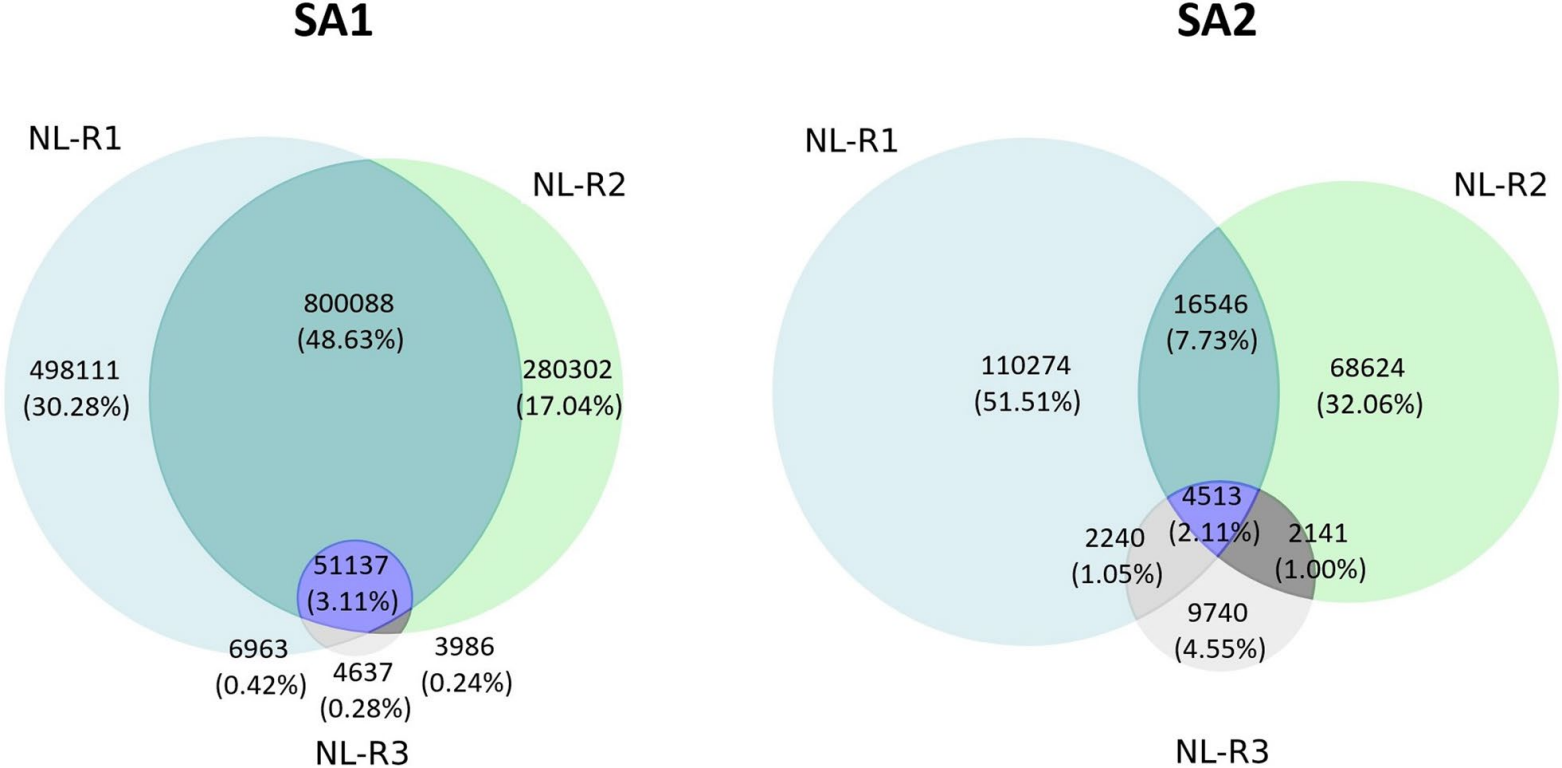
Illustration of the number of common sequences between the naïve library and different rounds of amplification for SA1 and SA2. In addition to the number of common sequences between the different datasets, the related percentages of sequences are also stated in parentheses

To investigate the heterogeneity of the two libraries in sequence composition, the number of common sequences in the naive library and different rounds of amplification was calculated for both experiments. Initially, it was found that no sequences were common between both lots, highlighting the large extent of heterogeneity of the two lots. In Fig. 4, the number of overlapping sequences for the different datasets of the two experiments is shown. As indicated in the Venn diagrams, for both lots there is a remarkable reduction in the number of common sequences between the naive library and consecutive amplification rounds shown by comparison at two levels as follows: NL-R1 (498,111) > NL-R2 (208,302) > NL-R3 (4,637) for SA1 and NL-R1 (110,274) > NL-R2 (68,624) > NL-R3 (9740) as well as NL-R1$\_$NL-R2 (800,088) > NL-R2$\_$NL-R3 (3986) for SA1 and NL-R1$\_$NL-R2 (16,546) > NL-R2$\_$NL-R3 (2141) for SA2, which highlights homogenization of the phage pools during amplification. The numbers in parentheses represent the number of common sequences between the datasets that are compared together. The two lots display substantial differences in decreasing number of common sequenecs during amplification at both levels of the above-mentioned comparisons as well as the number of common sequences among NL-R1$\_$NL-R2$\_$NL-R3 which is 51,137 for SA1 and 4513 for SA2. The increased homogeneity of phage populations can be attributed to the elimination of clones from the pool that is driven by amplification.

### The distribution of enrichment factors of peptides becomes more divergent during amplification with substantial discrepancies between lots

**Fig. 5:**
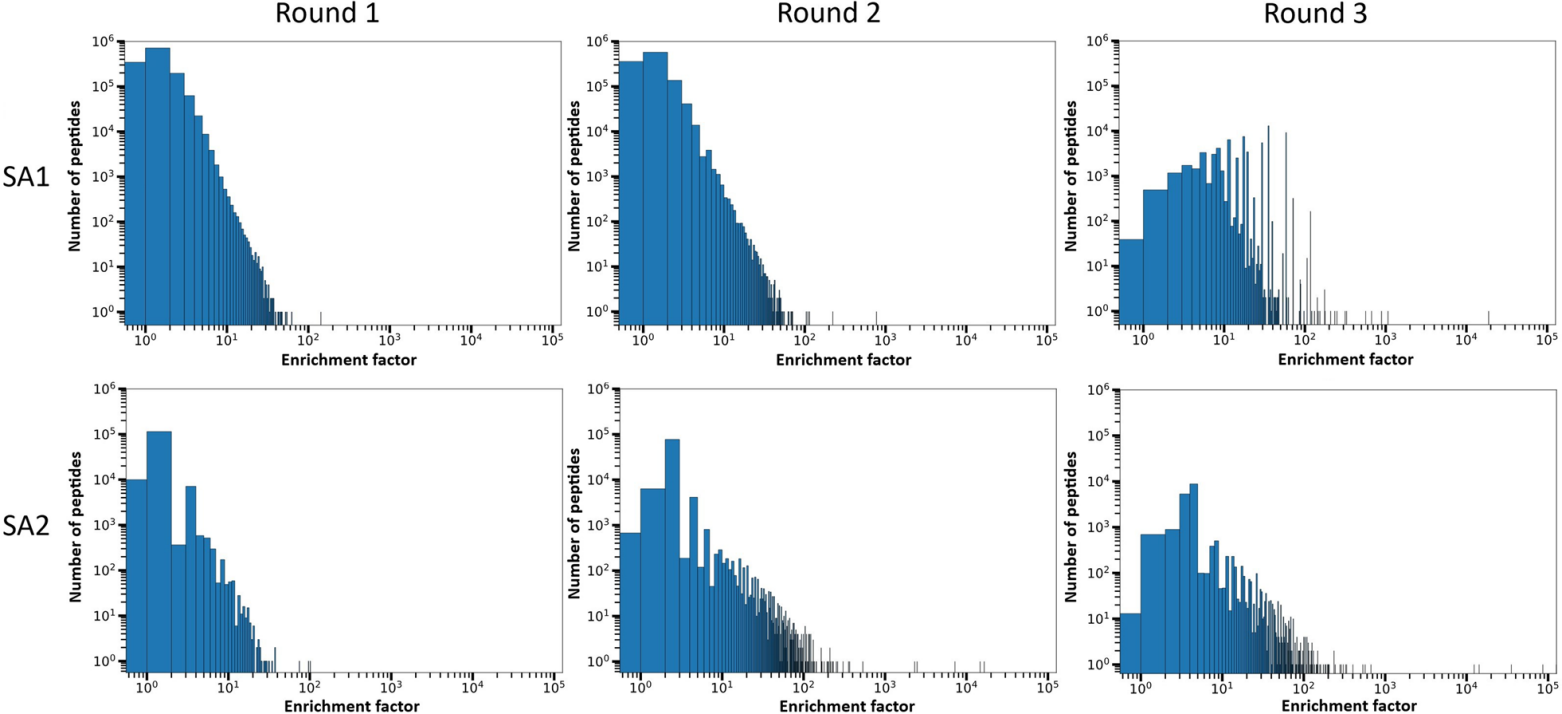
Distribution of enrichment factors (EFs) during the different rounds of amplification (Round 1, 2, and 3) for different lots of the peptide phage display library (SA1 and SA2). The horizontal axis represents the EF of peptides and the vertical axis represents the number of peptides having the given EF

To monitor the enrichment profile of sequences during serial amplification of the phage library, EF was calculated for the peptides. Using EF, peptides with actual enrichment during rounds of amplification can be identified. Relying on the absolute copy number (frequency) of peptides during amplification rounds can be misleading, as these peptides might also have a high frequency in the naïve library. Peptide sequences with EFs > 1 have been enriched during amplification, whereas peptide sequences with EFs = 1 have not been enriched during amplification and peptide sequences with EFs < 1 have been de-enriched during amplification. Hence, in the quest of identifying Pr-TUPs, which are peptides displayed on clones with increased propagation capacity, a criterion of EF > 1 should be fulfilled. To evaluate the distribution of EFs in each amplified pool, plots were made showing EFs and the number of peptides having each EF (Fig. 5). Generally, we observe an increase in the EF values for both lots during rounds of amplification. For instance, the highest EFs in rounds 1 and 3 are 142.86 and 18,861.05 for SA1, whereas the highest EFs in rounds 1 and 3 are 101.77 and 126,105.90 for SA2. This enhancement in the EF values is accompanied by a decrease in the number of peptides with lower EFs and an increase in the number of peptides with higher EFs. The phage pool consists of a subgroup of peptide sequences with very high EF values in round 2 and more pronounced in round 3 for both lots. These can be considered as potential Pr-TUPs. The list of the top 1000 peptide sequences from round 3 of amplification of both lots is available in Supplementary Table A4. For both lots, the number of peptides with EFs > 10^2^ notably increased in round 3 (from 1 sequence in round 1 of both lots to 199 sequences in round 3 of SA1 and 62 sequences in round 3 of SA2). Also, it is evident that with increasing rounds of amplification, the distribution of EFs becomes more diverse. This is clear from the broadening of the EF values in rounds 2 and 3 compared to the more compact pattern in round 1. This increasingly diverse distribution of EF values highlights a more heterogeneous distribution of peptide frequencies, which is caused by the remarkable enrichment of fast-propagating phage clones in the later rounds of amplification. During rounds of amplification, the phage pool diversity collapses resulting in a lower number of distinct phage clones but with notably higher propagation rates compared to the other clones of the library.

When comparing the two lots, the distribution of EFs is substantially different. The most prominent difference is observed in round 3, which indicates with increasing rounds of amplification a higher divergence can be observed between the two lots. Consistent with the findings shown in Fig. 4, the most enriched sub-population in each of the two lots has no sequences in common (Supplementary Table A4).

### Global and positional frequencies of amino acids indicate distinct compositional changes between lots and during amplification

The amino acid composition of the most enriched fraction (top 1000 peptide sequences with the highest EF values) of phage pools was investigated by calculating the observed global and positional frequencies of amino acids. The expected amino acid frequencies were calculated based on the number of available codons (according to the NNK randomization strategy used for library construction) for each amino acid.

**Fig. 6:**
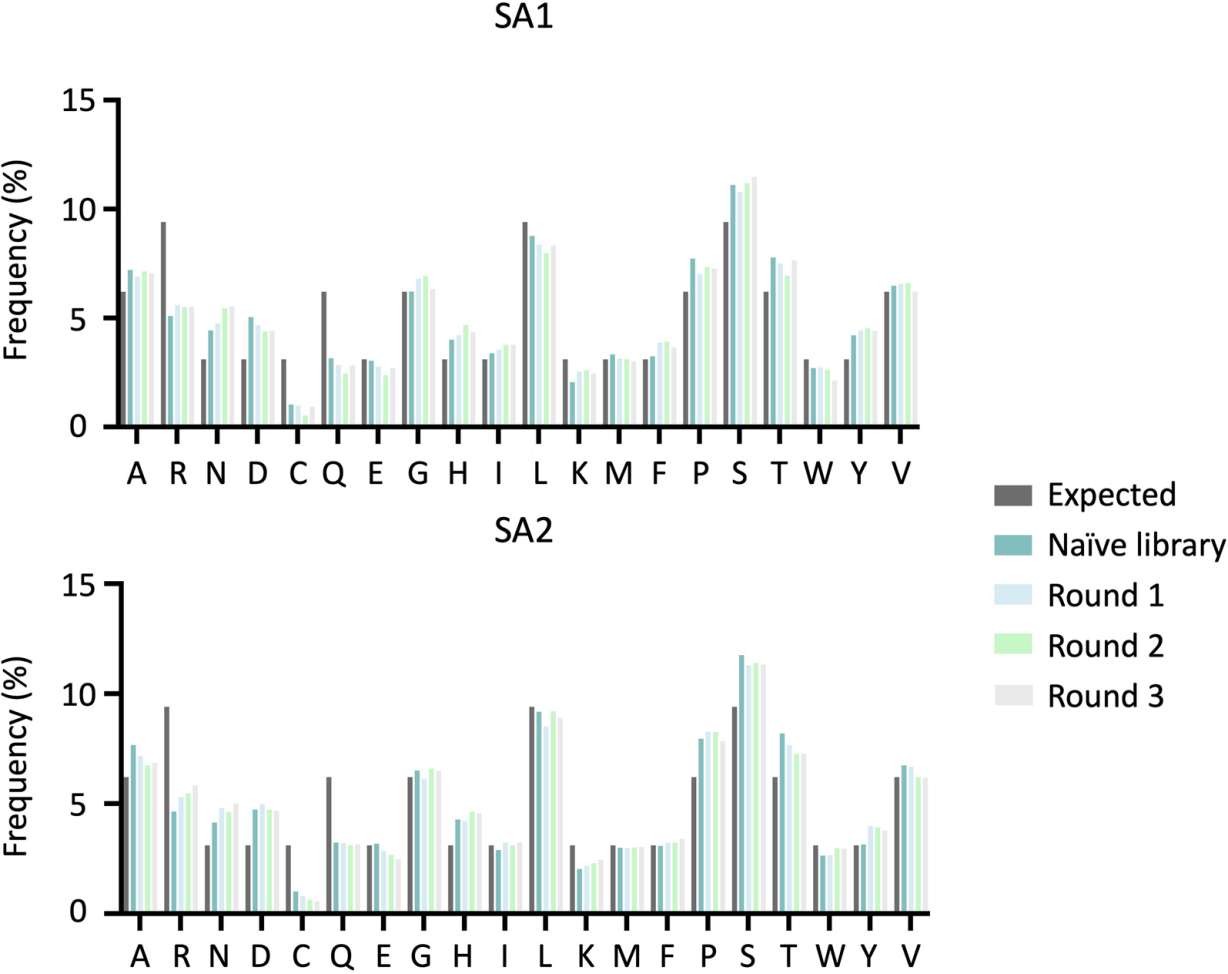
Global frequency for the top 1000 peptide sequences ranked according to EF for both SA1 (upper graph) and SA2 (lower graph). The global frequencies are shown for the expected (theoretical) frequencies of amino acids as well as for the observed frequencies of amino acids in the naïve library and the three rounds of amplification (Round 1, Round 2, and Round 3)

The global amino acid frequencies were determined for the naïve library and the amplification rounds of both lots. The observed global frequencies for each amino acid were calculated for every sample and compared to the expected frequencies (Fig. 6). It was found that the most common amino acid is serine, and the least common amino acid is cysteine in both lots. Substantial differences can be observed when comparing the expected frequencies for arginine, asparagine, aspartic acid, cysteine, glutamine, histidine, lysine, proline, serine, threonine, and tyrosine to the observed frequencies in naïve library and rounds of amplification. Once comparing the amino acid frequencies in amplification rounds to the naïve library, some similarities can be observed between lots, such as overrepresentation of arginine, asparagine, and lysine as well as underrepresentation of cysteine and glutamic acid. However, the two lots also indicate some discrepancies in the amino acid frequencies during amplification, such as overrepresentation of phenylalanine, as well as underrepresentation of aspartic acid, glutamine, proline, and tryptophan in SA1, but overrepresentation of tryptophan and tyrosine as well as underrepresentation of alanine and serine in SA2.

**Fig. 7:**
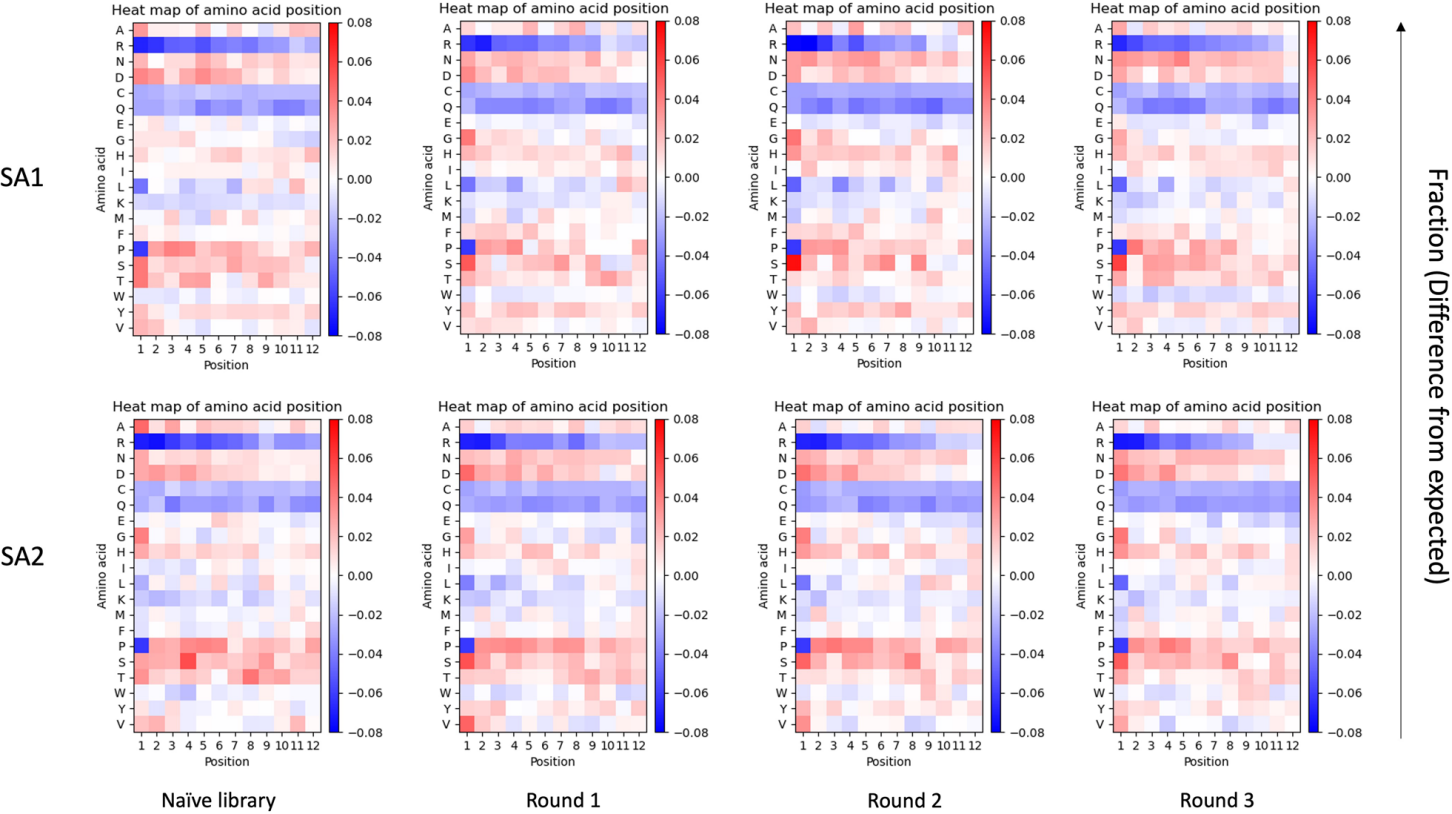
Positional frequency for the top 1000 sequences ranked according to EF for both SA1 (upper row) and SA2 (lower row). For each amino acid in the 12 different positions, the difference from the expected value was calculated and determined as a fraction. Red: overrepresented compared to the expected value, blue: underrepresented compared to the expected value

Subsequently, the positional frequencies of amino acids (on each of the 12 positions throughout the displayed peptide sequence) were investigated for the naïve library and rounds of amplification (Fig. 7). For the naïve libraries from both lots, we observe a non-random pattern of amino acids, before being subjected to any selective pressure for amplification. We observe an overall underrepresentation of arginine, cysteine, and glutamine, and an overall overrepresentation of alanine, asparagine, aspartic acid, histidine, proline (except position one), serine, and threonine in the naïve libraries of both lots. However, there are some dissimilarities between the naïve libraries from the two lots, e.g., alanine and histidine show a higher representation in SA2 compared to SA1, whereas isoleucine, phenylalnine and tyrosine show a lower representation in SA2 compared to SA1. This discrepancy in the naïve library will lead to differences in the amino acid composition in the amplified pools of the two lots. During rounds of amplification, several amino acids converge to a similar pattern in both lots, for instance, the presence of arginine increases in the last positions compared to the beginning of the peptide, where it is most underrepresented. Furthermore, leucine at position one becomes more underrepresented, whereas glycine and serine at position one become more overrepresented with rounds of amplification.

Furthermore, comparison of the plots of amplified libraries from the two lots indicates lot-specific patterns of amino acid representation at many positions; e.g. position 5 of alanine, position 4 of asparagine, position 10 of aspartic acid, position 12 of glutamic acid, position 2 of glycine, position 6 of leucine, position 1 of phenylalanine, position 5 of proline, position 10 of tryptophan, position 5 of tyrosine, etc. As a whole, our findings reveal major differences in the amino acid composition of the most enriched peptides of the two lots and highlight different sequence-dependent evolution of these lots during rounds of amplification.

### Motif analysis led to the identification of some motifs with probable association with a higher amplification rate of phage clones

**Fig. 8:**
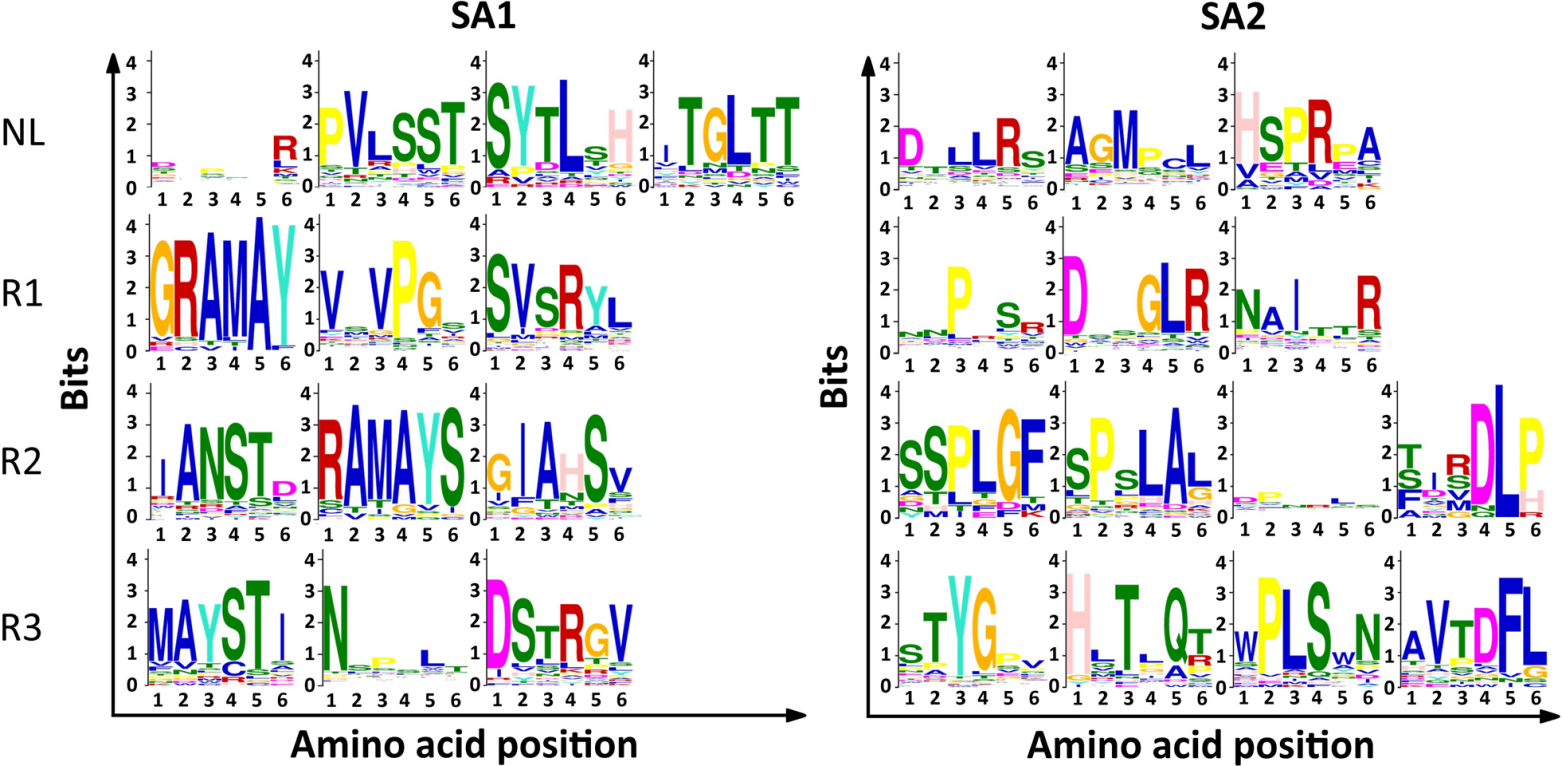
The discovered hexapeptide motifs from the STREME analysis on the top 18,622 sequences (ranked based on EF) of the naïve library (NL), round 1 (R1), round 2 (R2), and round 3 (R3) of both SA experiments. The discovered tripeptide motifs can be seen in Supplementary Fig. A1

Motif analysis was performed to discover motifs, in the primary and secondary structure of peptides, which might be associated with higher amplification rate of phage clones. Motif discovery in the primary structure of peptides was conducted through the STREME tool in the MEME database to identify ungapped tripeptide and hexapeptide motifs in the naive library and in the samples from amplification rounds. Only sequences with EF > 1 were included in the analysis to restrict motif identification to the most enriched sub-population of peptides in the pools. These highly enriched peptides are more likely to represent Pr-TUPs. The amino acid lengths were selected because a three-amino acid sequence is known as the minimal length for a functional motif [34] and a six-amino acid sequence is the average length of SLiMs [35], which are short linear functional motifs found in nature. A search for motifs in the naïve library was performed as a measure to identify background motifs (i.e., motifs with no association with amplification). The motifs identified by STREME are shown in Fig. 8 (hexapeptide motifs) and in Supplementary Fig. A1 (tripeptide motifs). To obtain a deeper understanding of the probable contribution of these motifs to increased amplification, the number of reads containing the identified motifs was determined and an enrichment score (ES) for each motif during amplification rounds was calculated. ES was calculated as the ratio between the number of reads containing the motif in the given round of amplification and the number of reads containing the motif in the naïve library (Table 1 and Supplementary Tables A5 and A6). A higher ES increases the probability of a motif being correlated with an increased amplification rate.

The results of the motif analysis indicate that except for a few identical motifs (like DLR, WPL, VPV, GLR), most of the motifs differ between the lots (Fig. 8 and Supplementary Fig. A1). There are many dipeptide motifs that are likely false-positive hits stochastically found in the peptide pools. Furthermore, many of the identified motifs are present in a low number of unique reads. This is obvious for hexapeptide motifs that are found in a lower number of unique reads compared to tripeptide motifs (Supplementary Tables A5 and A6). There are some tripeptide motifs that are found in a remarkably high number of unique sequences (like LPL, SLP, LPS, DLR, among others). However, they all possess low ES. The only exception is the motif MAY which has the highest ES among all tripeptide motifs.

Most of the hexapeptide motifs also possess low ES, but a few motifs including SVSRY, GRAMAY, RAMAYS, MAYSTI exhibit high ES (Table 1 and Supplementary Table A6). Interestingly, three of these sequences harbor the penta-amino acid motif RAMAY that is conserved in rounds 1 and 2, and the tetra-amino acid motif MAYS that is conserved in rounds 2 and 3 of SA1. The tri-amino acid motif MAY has been conserved through all rounds of amplification. The conservation of these amino acid sequences during rounds of amplification as well as their absence in the naïve library strengthens the hypothesis that these motifs might genuinely contribute to the increased amplification rates of the respective phage clones.

**Table 1:** Enrichment score (ES) of the discovered hexapeptide motifs from the STREME analysis during amplification rounds

	Motifs	R1	R2	R3
		ES		
SA1	DLPSLR	1.00	1.50	0.00
	PVLSST	1.00	1.00	0.00
	SYTLSH	1.89	3.20	0.11
	ITGLTT	0.00	0.00	0.00
	GRAMAY	11.1	13.3	0.49
	VSVPGS	2.33	1.67	0.00
	SVSRYL	10.5	9.75	0.25
	IANSTD	0.00	0.00	0.00
	RAMAYS	11.1	13.3	0.49
	GIAHSV	5.00	4.00	0.00
	NSPSLT	4.00	1.00	1.00
	DSTRGV	1.00	0.33	0.33
	MAYSTI	11.1	13.3	0.49
SA2	DTLLRS	0.00	0.00	0.00
	AGMPCL	0.00	0.00	0.00
	HSPRPA	0.00	0.00	0.00
	NNPRSR	1.00	1.00	0.00
	DSSGLR	1.25	1.75	0.25
	NAITTR	2.00	1.00	0.00
	DPNRLS	2.00	7.00	7.00
	TIRDLP	0.00	0.00	0.00
	SSPLGF	2.00	3.00	0.00
	SPSLAL	0.00	0.00	0.00
	STYGPV	0.00	0.00	0.00
	HLTLQT	1.00	0.00	1.00
	WPLSWN	0.00	0.00	0.00
	AVTDFL	0.00	0.00	0.00

See Supplementary Table A5 for further information on tripeptide motifs and Supplementary Table A6 for further information on hexapeptide motifs. R1: Round 1, R2: Round 2, R3: Round 3

We tried to add a new dimension to motif analysis through motif search in the amino acid sequences associated with the secondary structure of the peptides. This analysis was based on finding motifs previously known to be associated with $\beta$-turn formation [36, 37]. The presence of a $\beta$-turn in the displayed peptide, in particular at its N-terminus, has already been reported to result in an increased amplification rate of the respective phage clones [26, 38]. Similarly to the previous analyses, we determined the number of peptides containing each of the motifs associated with $\beta$-turn formation and calculated ES for each motif during amplification (Table 2 and Supplementary Table A7). We found that these motifs are present in a low number of reads and they all show low ES. Investigation of the location of these motifs in the peptides also indicated that a very low number of peptides contain these motifs at their N-termini. Overall, our observations highlight the lack of correlation between these $\beta$-turn-associated motifs and the increased amplification rate of phage clones.

**Table 2:** Enrichment score (ES) of the eight $\beta$-turn related tetrapeptide motifs during amplification rounds of both SA experiments

	Motifs	R1	R2	R3
		**ES**		
SA1	PPNP	1.45	1.16	0.051
	DPGG	1.36	0.89	0.034
	DPDG	1.45	1.04	0.053
	DPNG	1.99	1.58	0.090
	YGNT	1.42	0.92	0.021
	PNWN	1.41	1.03	0.038
	PPGC	1.28	0.32	0.015
	YNGK	1.46	1.03	0
SA2	PPNP	1.13	0.74	0.087
	DPGG	1.14	0.43	0.095
	DPDG	1.00	0.85	0.154
	DPNG	1.08	1.54	0.154
	YGNT	1.00	0.86	0.143
	PNWN	1.33	2.50	1.167
	PPGC	1.17	0.33	0
	YNGK	1.00	0	0

See Supplementary Table A7 for further information on these motifs. R1: Round 1, R2: Round 2, R3: Round 3

## Methods

### Phage display peptide library

Two different lots of the Ph.D.™-12 library (New England Biolabs, Ipswich, Massachusetts, United States of America, lot no.: 10111202, and lot.no.: 10122218, cat. no.: E8111LVIAL) were used for serial amplification. The M13KE phage (a derivative of the M13mp19 vector) has been used to construct this library. The manufacturer (New England Biolabs) has reported the complexity of this library to be 10^9^ sequences fused in-frame to the pIII (minor coat protein) on the M13 phage. The peptides are expressed at the N-terminus of pIII, meaning that the first randomized position is the first residue of the peptide-pIII fusion protein. A Gly-Gly-Gly-Ser spacer is inserted between the displayed peptide and pIII. Each peptide is expressed in five copies on the surface of each virion.

### Serial amplification of the library and phage extraction

For each lot of the phage display peptide library, two replicates were serially amplified for three consecutive rounds. The *Escherichia coli* (*E. coli*) F^+^ strain ER2738 (New England Biolabs, Ipswich, Massachusetts, United States of America) was used for the amplification of phage pools. The genotype of the bacterial strain is F′ *proA*^*+*^ *B*^*+*^ *lacl*^*q*^ △*(lacZ)M15 zzf::Tn10*(Tet^R^)*/ fhuA2 glnV* △*(lac-proAB) thi-1* △*(hsdS-mcrB)5*. This strain has a rapid growth rate and is tetracycline-resistant. An overnight culture of ER2738 in lysogeny broth (LB) medium containing tetracycline (20 μg/mL; Merck, Darmstadt, Germany) was diluted in a fresh LB medium. The bacterial culture was infected with 10^9^ phages at the early log phase (OD600 ≤ 0.2), and incubated at 37°C, 250 rpm, 5 h, yielding similarly low multiplicity of infection (MOI) for both lots. The low MOIs increase the efficiency of phage amplification and avoid biased competition among phage clones, associated with a low number of bacterial cells.

After amplification, the bacterial cells were removed by centrifugation (5000× g for 15 min at 4°C). Phages were extracted by adding 1/6 volume of PEG/NaCl buffer (20% PEG/2.5 M NaCl) and incubated at 4°C overnight. The next day, phages were pelleted by centrifugation at 12,000 g for 20 min at 4°C and resuspended in 1 mL of Tris Buffered Saline (TBS).

### Phage titration

Phage titering was performed to enumerate phage particles in the naïve and amplified phage pools and conducted by the double agar overlay method. This approach enables us to determine the number of infective phage particles. The phage samples were serially diluted (10-fold) in LB medium, added to 200 μL of mid-log ER2738 culture (OD600 ≥ 0.5), and allowed to infect the bacterial cells for 5 min at room temperature (RT). The infected bacterial cells were added to 3 mL of top agar (0.7% agar) and plated on LB agar plates containing tetracycline, 5-bromo-4-chloro-3-indolyl-*β*-D-thiogalactopyranoside (X-gal) (Thermo Fisher Scientific, Waltham, Massachusetts, United States of America) and isopropyl *β*-D-thiogalactopyranoside (IPTG) (Thermo Fisher Scientific, Waltham, Massachusetts, United States of America). Plates were inverted and incubated at 37°C overnight. Blue plaques, each representing a single phage clone capable of infecting the host bacterium, were counted and used for calculation of the titer of phage samples (pfu/mL). The titration of each phage sample was done in triplicates.

### Sample preparation for Illumina sequencing

Single-stranded DNA (ssDNA) was purified from the naïve library and amplified phage pools by NucleoSpin® Plasmid, Mini kit for plasmid DNA (Macherey-Nagel, Düren, Germany) using an adapted version of the manufacturer’s supplementary protocol (NucleoSpin® Plasmid - isolation of M13 DNA omitting the amplification step). The purified DNA was eluted in RNase-free water (Qiagen, Hilden, Germany). The first PCR reaction was performed to amplify the target sequence in the phage genome, which contains the variable region that encodes the displayed peptide. In addition, this PCR was aimed to obtain amplicons with adapters at both ends to make them compatible with the Illumina sequencing platform. The following primers were used for target sequence amplification.

Forward primer: 5′-TCG TCG GCA GCG TCA GAT GTG TAT AAG AGA CAG ACC TCG AAA GCA AGC TGA TAA ACC G-3′

Reverse primer: 5′-GTC TCG TGG GCT CGG AGA TGT GTA TAA GAG ACA GCT GTA GCA TTC CAC AGA CAG CCC-3′

These primers contain phage genome-specific nucleotides, which are bound to sites in the phage genome flanking the variable region, and adapters. The underlined nucleotides represent the overhang adapter sequences.

The PCR reaction was performed in 50 μL volumes (2.5 μL of 10 μM forward primer, 2.5 μL of 10 μM reverse primer, 25 μL of Q5 High-Fidelity 2X Master Mix (New England Biolabs, Ipswich, Massachusetts, United States of America), 100 ng of template, and RNase-free water. The following PCR program was used: initial denaturation (98°C for 30 s), 10 cycles of denaturation at 98°C for 10 s, annealing at 60°C for 30 s, and extension at 72°C for 30 sec followed by a final extension at 72°C for 2 min. The PCR products were purified using the QIAquick PCR Purification Kit (Qiagen, Hilden, Germany), and 25 ng was used as input for the index PCR. The purpose of this PCR was to add indices to the amplicons, which allowed for multiplexed sequencing. The cyclic conditions for the index PCR were as follows: initial denaturation at 95°C for 3 min, 8 cycles of denaturation at 98°C for 30 s, annealing at 60°C for 30 s, extension at 72°C for 30 s, and final extension at 72°C for 5 min. The PCR products were purified using the QIAquick PCR Purification Kit (Qiagen, Düren, Germany) and eluted in RNase-free water.

### Illumina sequencing

Sample﻿s underwent quality control at the Center for Genomic Medicine, Copenhagen University Hospital, Rigshospitalet (Copenhagen, Denmark) using the Fluoroskan™ Microplate Fluorometer (Thermo Fisher Scientific, MA, USA) by the Quant-iT™ 1X dsDNA HS Assay (Thermo Fisher Scientific, MA, USA). Samples were sequenced by single-end sequencing using the NextSeq Sequencing System (Illumina, USA) and the Mid Output Kit v2.5 (300 Cycles) (Illumina, USA) with a read length of 250 base pairs.

### Analysis of NGS data

Demultiplexing of the base call (BCL) file into individual FASTQ files was performed using the bcl2fastq software provided by Illumina. The FASTQ files underwent quality filtering using a Python script. Initially, the script removed reads with three nucleotides with a Phred score below Q20 to reduce the number of reads with sequencing errors. Then, the nucleotides were translated into amino acids. The script further removed reads without the GGGS-linker (i.e., wildtype sequences) and reads containing ambiguous nucleotides (i.e., ‘X’ or ‘*’). The FASTQ files from different rounds of amplification were combined into files containing the sequences of all unique reads, the number of reads, relative frequency, and the calculated enrichment factor (EF) (i.e., naïve library- round 1, naïve library-round 2, etc.). The EF was calculated as the ratio between the relative frequency of the peptide sequence in one of the rounds of amplification and the relative frequency of the same peptide sequence in the naïve library for both replicates. EF provides a measure of how much the sequence is enriched during rounds of amplification while taking into account the abundance of the peptide in the experimental input. Sequences with EF = 1 were considered to have no enrichment, whereas sequences with EF < 1 were considered to be de-enriched during amplification and sequences with EF > 1 were considered to be enriched. Furthermore, the script combines the files from different rounds of amplification with the naïve library.

For each experiment, two replicates were included. The two replicates were merged, and in the case of duplicate sequences, the copy of each duplicate sequence with the lower EF was removed to include any potential clones of interest from subsequent EF-based analyses. For each round, a file combined with the naïve library was generated (naïve-round 1, naïve-round 2, etc.).

### Bioinformatic analyses

The merged files were used to calculate the percentage of wildtype clones and the number of unique sequences as well as for the preparation of stacked bar plots. For other analyses, sequences with EF values were included as follows: all sequences with EF value for the identification of common sequences among samples and EF distribution, the top 1000 in the EF-ranked datasets for the calculation of global and positional frequencies of amino acids, and the top 18,622 sequences in the EF-ranked datasets for motif analysis.

To investigate the frequency-based distribution of peptides and gain insights into diversity changes within each peptide population, each sample was grouped into bins (sub-populations) according to their copy number (abundance) using a MATLAB script. The number of sequences in each bin and the sum of relative abundances were calculated. The stacked bar plots were generated showing the number of unique sequences as well as the sum of abundances in each of the bins. The stacked bar plots were made to compare the size of the peptide sub-populations between samples.

The number of common sequences between all peptide pools (naïve library and amplification rounds) for both SA experiments was shown with Venn diagrams. They illustrate the overlap of common unique sequences between different datasets and note the correlated number of sequences of that area with the related percentage value. The Venn diagrams were created using the Matplotlib 2D graphics package matplotlib-venn version 3.5.2 for Python [39].

The general distribution of EF values for all rounds of each SA experiment was examined by binning the EF value population into intervals of 1. This was then visualized as a histogram with EF values on the x-axis and the number of sequences with the correlated EF value on the y-axis. The histograms were created using the Matplotlib 2D graphics package matplotlib.pyplot for Python [39].

To characterize the amino acid composition of peptide pools, the global and positional frequencies of individual amino acids were determined. The global and positional frequencies were determined by using the top 1000 peptide sequences ranked based on their EF values. The global and positional frequencies were compared to the theoretically expected values. The expected values were calculated based on the number of available codons for each amino acid according to the NNK codon randomization used for library construction. Motif analysis was performed on both the primary and secondary structures of the most enriched sub-population of peptide pools. Motif analysis on the primary structure of peptides was performed using the motif discovery tool STREME [40]. This motif discovery tool identified ungapped tripeptide and hexapeptide motifs enriched compared to a shuffled dataset of the input sequences until three consecutive motifs were discovered with a p-value exceeding 0.05. The statistical test was a Fisher Exact Test. The input dataset consisted of the top 18,622 sequences (based on EF value) in FASTA format. This number of sequences was chosen as it allowed for equal treatment of all samples, being the highest number that could be extracted from each sample while remaining within the maximum limit that could be run. The discovered motifs were subsequently used to identify all motif-containing sequences in the naïve library and amplification rounds of both SA experiments. Finally, the secondary structure of peptides was investigated for the presence of eight reported tetrapeptide motifs associated with *β*-turn formation in the naïve library and amplification rounds of both SA experiments. All sequences with an EF > 1 were included in this analysis. If sequences contained one of the *β*-turn-related motifs, the analysis also noted if the motif was located at the N-terminus of the corresponding peptide.

## Discussion

It is critically important to investigate the composition of phage display libraries to increase the likelihood of a successful ligand discovery. Gaining insights into the peptide content of phage pools can help in the development of successful selection strategies for finding target-specific ligands. On the other hand, amplification is a central component in phage display protocols; however, it is well known that amplification generates bias in the phage pools. Therefore, knowing how amplification can alter the sequence composition of phage display libraries is essential. To our knowledge, this is the first report based on deep sequencing by NGS in which the peptide composition of different lots of the same naïve phage display library is investigated and their compositional features are monitored during three successive rounds of amplification. Additionally, we included two replicates for amplified phage pools to cover a broader diversity and for increased accuracy in our analyses.

In the current work, a significant heterogeneity between peptide compositions in the two lots was found. This was highlighted by observing no common sequences between the two lots in the analysis of overlapping sequences (Fig. 4). The observed heterogeneity between the two lots might have several explanations. First and foremost, it can reflect the actual heterogeneity present in the library. This arises during the library construction, as the complete sequence space cannot be covered by the library due to a limited transformation efficiency of phage vectors into the host bacterial cells. The theoretical sequence space of a 12-mer library is 4.1 × 10^15^ and taking the maximal complexity 10^9^ that can be achieved during transformation, the Ph.D.™-12 library solely covers 0.00002% of all possible sequences in the optimal scenario. Another explanation is the sampling or preparation bias, as there are several steps in the experiments in which bias can happen. This type of bias can take place both before sequencing once the samples are prepared for NGS (withdrawal of phages from the naïve library or amplified phage pools, DNA extraction from phages, PCR amplification, purification of PCR products, and indexing) and during sequencing of amplicons (loading a subset of phage-derived DNA onto the flow cell, amplification during sequencing, and the occurrence of sequencing errors). However, this is a less likely scenario, as no common sequence was found even in the naïve libraries of both lots. Due to the larger diversity compared to amplified phage pools, naïve libraries are less prone to sampling and preparation bias. Another less probable explanation is the restriction of the currently used NGS platforms which only cover a minor fraction of the whole sequence space present in the library. We also observed a decreased number of common sequences between the naïve library and amplified pools with the progress of amplification (Fig. 4). Reduction in the number of common sequences between the naïve library and rounds of amplification for each lot can be attributed to the competitive evolution of the pool under amplification pressure and, thus, the consequential elimination of many clones from the library. This pattern is observed in both lots; however, with some variations, suggesting that the two lots have different evolutionary trajectories. Heterogeneity between the two lots is also illustrated by discrepancies in the number of unique sequences and the percentage of wildtypes (Fig. 1), as well as dissimilarities in diversities shown by the different percentages of singletons and repeated sequences between the two lots (Fig. 3) where the entire peptide pool is included in the analysis. This heterogeneity is also reflected by differences in the distribution of EFs (Fig. 5), and the distinct patterns of amino acid frequencies (Figs. 6 and 7) when using datasets sorted based on EFs.

To investigate the diversity of phage pools, the number of distinct sequences was calculated. A decrease in the diversity of phage display libraries during amplification has been reported previously [31, 41]. In the present work, the reduced diversity of the phage display libraries was observed by the reduced number of unique sequences and the increased percentage of wildtypes (Fig. 1). As seen in the figure, the number of unique sequences and the abundance of wildtypes are inversely correlated, and it can be seen for both lots that the largest increase in the wildtype frequency and the biggest collapse in the number of unique sequences occur in round 3. This might underscore that successive rounds of amplification can be avoided for library selection, as it leads to an increase in the wildtype percentage in the resulting phage pool. In the case of a biopanning sample, the percentage of wildtypes might increase even more, as it is likely that there are fewer competitors for wildtype clones in the phage pool. This is also in line with evidence from the literature highlighting that only one round of selection might be sufficient for the enrichment of target-binding ligands by using NGS [21]. Our findings also revealed lot-specific patterns in the diversity changes.

In the analysis of EF distribution across the two experiments during different rounds of amplification (Fig. 5), a trend of an enhancing number of fast-propagating clones (i.e., clones with higher EFs) and increasing EFs were observed for both lots. However, the heterogeneity between the two experiments is highly evident in this analysis, showing a similar trend, but with SA1 containing a larger pool of peptides with increased EFs. One of the major differences can be observed for round 3, where SA1 contains a higher number of peptides with high EFs and a broader distribution of different EFs compared to SA2. The global and positional frequencies of the individual amino acids were determined among the top 1000 peptide sequences, ranked according to their EFs (Figs. 6 and 7). The positional frequency provides additional insight into the pool of sequences, as the analysis can show differences in frequencies in a sequence-dependent context, enabling the identification of amino acid contents that are associated with amplification. The frequency patterns (under- and overrepresentation of certain amino acids in the amplified pools compared to the naïve library) observed in the amplified samples in this analysis might be potential indicators of a higher amplification rate.

The bias associated with amplification can be either sequence-dependent or sequence-independent [27]. Sequence-independent bias results from mutations within the phage genome that increase the amplification rate of phage clones, irrespective of the displayed sequence. Thus far, point mutations [28], genomic rearrangement [42], and genomic deletion [29] have been described as causes for higher amplification rates of phage clones. Sequence-dependent bias has not been extensively examined. However, it has been found that some sequences infer a lower amplification rate of phage clones, and as a result, such sequences are under-represented in the library [38]. On the other hand, little is known about sequences that increase the amplification rate of phage clones [43]. In line with this, we attempted to identify motifs that are likely associated with the increased amplification rate of phage clones. To rule out stochastically occurring motifs, the motifs had to meet some criteria. One criterion was that those sequences with the highest EFs (a dataset with the top 18,622 sequences ranked based on EF) were included in the analysis. Additionally, motif analysis was performed on the naïve library as well, as a measure of identifying background motifs, which are not likely to be associated with a higher propagation capacity. Taking the vastly different compositions of the two lots into consideration, a similar motif between the two lots can bear valuable information about amplification. The length of the motifs was determined to be either three or six amino acids. A three-amino acid sequence is considered the minimal length of a motif [34], and a six-amino acid sequence is the average length of a short linear motif (SLiM) [35]. SLiMs are short, conserved motifs rich in the intrinsically disordered regions of the human proteome and play important roles in many protein-protein interactions in nature [44]. The motif search in the current work is not restricted to the findings of the STREME analysis but contains a greater investigation into the STREME output to provide stronger evidence for the genuineness of the identified motifs. This was performed by calculating the number of absolute reads containing the given motif and calculating the ES for each motif during amplification. Our analysis led to the identification of some motifs with high ES (Table 1), which have been conserved during rounds of amplification (Fig. 1). These motifs are very likely to be associated with a higher amplification rate of the respective phage clones. However, they are not found in many of the highly frequent peptides (listed in Supplementary Fig. A4) that are displayed on the most enriched clones. This is due to the fact that higher propagation rate of these clones, which leads to their enrichment during amplification rounds, can also result from their genotypic features (e.g. propagation-enhancing mutations in the phage genome). Thus, the genetic mapping of mutations in the most abundant clones can offer more detailed insights into the mechanisms driving the propagation of these clones. The motif analysis was further extended to the secondary structure of peptides, where motifs associated with the formation of $\beta$-turns were searched for. It has been found that peptides with a $\beta$-turn amplify quicker than peptides containing an $\alpha$-helix, especially if the $\beta$-turn is positioned at the N-terminus of the displayed peptide [38]. However, given the low ES and scarce number of sequences containing the motifs associated with $\beta$-turns (Table 2 and Supplementary Table A4), no correlation was found. In our work, the data are based on the analysis of a large dataset obtained from NGS of the phage pools, unlike the previously mentioned report that relies on the analysis of a limited number of peptide sequences by Sanger sequencing of the phage pools.

The emergence and undesirable isolation of TUPs in the biopanning outputs is very common and can be considered as the major issue associated with peptide phage display. Identification and reporting of Pr-TUPs hold great promise for the phage display community to help elucidate the nature of identified peptide leads. We identified the top 1000 sequences with the highest EFs (Supplementary Table A4) from each lot of the Ph.D.™-12 phage display library, which is the most commonly used phage display peptide library for biopanning according to the statistics available through Biopanning Data Bank (BDB). Our findings reveal that Pr-TUPs are specific to each lot. This can be explained by the extensive heterogeneity between the two lots of the phage display library in question. Our list of identified peptides can serve as a control for the analysis of biopanning data when using these two lots of the library, as the isolation of these sequences in the selection output cannot be attributed to target-specific selection and these peptide sequences should be filtered out of the biopanning output.

## Conclusion

Understanding the peptide composition of the naïve and amplified phage display libraries can aid the search for promising target-binding ligands. The outcome of phage display selections can be affected by differences in the sequence composition of different lots of phage display libraries. In the current work, we investigated the composition of peptide pools at three different levels: the level of peptides, the level of amino acids, and lastly, the level of motifs. Our findings reveal substantial heterogeneity between different lots of the same phage display library, and additionally provide evidence for the different evolutionary trajectories of different lots of the library during amplification. This was observed by the considerable changes in the composition of the phage pool during the amplification of each lot and that these compositional changes are specific to each lot. The findings of the current study can be illuminating for phage display researchers by providing them with novel insights into the complexities of phage display peptide libraries and advancing their understanding of how these complexities can impact the interpretations of phage display selections.

## Supplementary Information

Supplementary file 1

## Data Availability

The raw data and scripts are available upon request

## Abbreviations

BDB: Biopanning Data Bank
DNA: Deoxyribonucleic acid
EF: Enrichment factor
ES: Enrichment score
E. coli: Escherichia coli
IPTG: Isopropyl $\beta$-D-thiogalactopyranoside
LB: Lysogeny broth
MOI: Multiplicity of infection
NEB: New England Biolabs
NGS: Next-Generation Sequencing
NL: Naïve library
P3: Protein 3 of the M13 phage
PEG: Polyethylene glycol
pfu: Plaque forming unit
Pr-TUP: Propagation-related target-unrelated peptide
R1, R2, and R3: Round 1, Round 2, and Round 3 of amplification
SA: Serial amplification
SLiM: Short linear motif
ssDNA: Single-stranded DNA
TBS: Tris-buffered saline
TUPs: Target-unrelated peptides
WT-M13KE: Library clone not expressing any peptide
X-gal: 5-Bromo-4-chloro-3-indolyl-$\beta$-D-thiogalactopyranoside
