# Supplementary material for: A comparative analysis of sequence composition in different lots of a phage display peptide library during amplification
Source: Virol J. 2025 Feb 1;22:24. doi: 10.1186/s12985-024-02600-x (PMC11786364; doi:10.1186/s12985-024-02600-x)
Supplement: Supplementary file 1 [file 12985_2024_2600_MOESM1_ESM.pdf]

Appendix A Supplementary Data

Table A1: Summary of NGS data for SA1 The numbers and percentages are shown for both replicates of each sample (NL: naïve library, Rep: replicate).

**Sample**	**Unique Cleaned Reads**	**Unique Removed Reads**	**Total Unique Reads**[1]	**Absolute Cleaned Reads**	**Absolute Removed Reads**	**Total Absolute Reads**[2]	**Percentage of Cleaned Unique Sequences**	**Percentage of Wild-type Clones**
NL	3,006,524	4,065,861	7,072,385	4,857,564	6,066,849	10,924,413	61.89	10.36
Round 1 Rep. 1	2,285,670	3,396,403	5,682,073	4,830,831	5,929,292	10,760,123	47.31	10.52
Round 1 Rep. 2	2,662,381	3,403,736	6,066,117	4,833,154	5,621,951	10,455,105	55.09	11.33
Round 2 Rep. 1	1,768,516	2,939,219	4,707,735	4,204,512	6,376,294	10,580,806	42.06	17.86
Round 2 Rep. 2	1,907,350	2,974,764	4,882,114	3,899,555	6,021,819	9,921,374	48.91	17.26
Round 3 Rep. 1	73,462	365,016	438,478	136,506	7,424,260	7,560,766	53.82	89.73

Continued on next page

**Sample**	**Unique Cleaned Reads**	**Unique Removed Reads**	**Total Unique Reads**[1]	**Absolute Cleaned Reads**	**Absolute Removed Reads**	**Total Absolute Reads** [2]	**Percentage of Cleaned Unique Sequences**	**Percentage of Wild-type Clones**
Round 3 Rep. 2	51,102	439,867	490,969	82,865	9,781,908	9,864,773	61.67	91.01

[1]Total unique reads are defined as the number of reads with different peptide sequences.
[2]Total absolute reads are defined as the total number of reads the whole sample contains.

Table A2: Summary of NGS data for SA2 The numbers and percentages are shown for both replicates of each sample (NL: naïve library, Rep: replicate).

**Sample**	**Unique Cleaned Reads**	**Unique Removed Reads**	**Total Unique Reads**[1]	**Absolute Cleaned Reads**	**Absolute Removed Reads**	**Total Absolute Reads**[2]	**Percentage of Cleaned Unique Sequences**	**Percentage of Wild-type Clones**
NL	2,102,225	3,977,930	6,080,155	2,174,815	4,467,971	6,642,786	96.66	5.99
Round 1 Rep. 1	1,261,141	3,365,136	4,626,277	1,305,929	3,905,364	5,211,293	96.57	7.63
Round 1 Rep. 2	1,234,625	3,096,092	4,330,717	1,278,422	3,601,161	4,879,583	96.57	8.62
Round 2 Rep. 1	851,181	2,587,361	3,438,542	1,040,891	4,172,500	5,213,391	81.77	24.46
Round 2 Rep. 2	720,000	2,189,286	2,909,286	939,916	3,815,570	4,755,486	76.60	27.16
Round 3 Rep. 1	100,077	709,070	809,147	581,582	5,139,007	5,720,589	17.21	64.65

Continued on next page

Sample	Unique Cleaned Reads	Unique Removed Reads	Total Unique Reads[1]	Absolute Cleaned Reads	Absolute Removed Reads	Total Absolute Reads[2]	Percentage of Cleaned Unique Sequences	Percentage of Wild-type Clones
Round 3 Rep. 2	159,634	892,702	1,052,336	498,047	4,313,283	4,811,330	32.05	58.97

[1]Total unique reads are defined as the number of reads with different peptide sequences.
[2]Total absolute reads are defined as the total number of reads the whole sample contains.

Supplementary Table A4 is available through this link: Supplementary Table A4

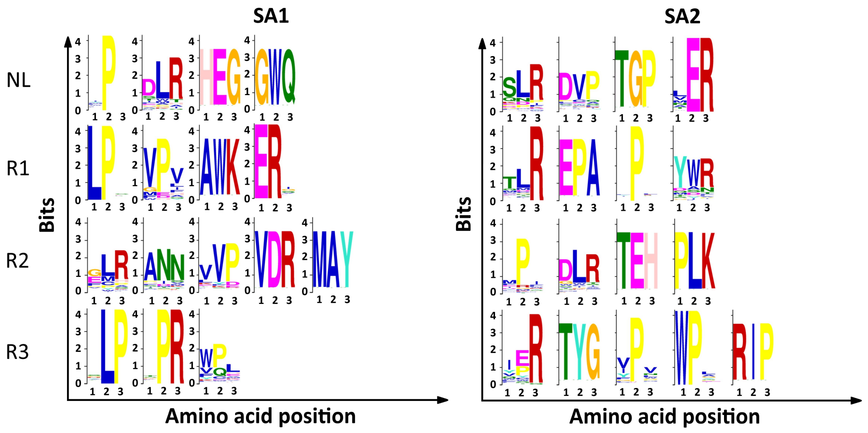

Fig. A1: The discovered tripeptide motifs from STREME analysis on the top 18,622 sequences (ranked based on EF) of the naïve library (NL), round 1 (R1), round 2 (R2) and round 3 (R3) for both SA experiments.

Table A3: Overview of changes in the distribution of peptide frequencies for the two experiments (SA1 and SA2) in the naive library and during amplification rounds. The samples were grouped into bins according to their abundance. Each bin was considered a population within the sample. The size of the population in percentage as well as the number of sequences in each population can be seen from the table. The percentages represent the sum of percentages in this population. c.p.: copy number.

SA1

	Unique sequences	Singletons (c.p. = 1)	Population 2 ($1 < \text{c.p.} \leq 200$)	Population 3 ($200 < \text{c.p.} \leq 5000$)	Population 4 ($\text{c.p.} > 5000$)
Naïve library	3006524	45.58%	52.41%	1.880 %	0.134 %
		2214080	792238	205	1
Round 1	1356299	16.05%	55.79%	20.52%	7.640%
		701483	653683	1106	27
Round 2	1135513	16.69%	46.20%	21.35%	15.75%
		644774	489687	1018	34
Round 3	66723	46.82%	30.96%	18.28%	3.941%
		61228	5470	24	1

SA2

	Unique sequences	Singletons (c.p. = 1)	Population 2 ($1 < \text{c.p.} \leq 200$)	Population 3 ($200 < \text{c.p.} \leq 5000$)	Population 4 ($\text{c.p.} > 5000$)
Naïve library	2102225	93.62%	6.385%	0%	0%
		2035960	66265	0	0
Round 1	133573	77.66%	21.56%	0.7779%	0%
		122693	10878	2	0
Round 2	91823	30.37%	24.78%	4.094%	40.76%
		83103	8693	24	3
Round 3	18634	3.255%	6.070%	1.216%	89.46%
		15329	3295	6	4

Supplementary Table A5. Overview of the number and percentage of unique sequences as well as the number and percentage of absolute reads containing the tripeptide motifs discovered by the STREME analysis in the naive library (NL), round 1 (R1), round 2 (R2), and round 3 (R3) of both SA experiments and the ES of these motifs during amplification rounds. Each column for each SA experiment has been color-weighed by a color gradient from low value with light green to high value with dark blue.

	NL				R1					R2					R3					
Motifs	Unique seq	Unique seq (%)	Reads	Reads (%)	Unique seq	Unique seq (%)	Reads	Reads (%)	ES	Unique seq	Unique seq (%)	Reads	Reads (%)	ES	Unique seq	Unique seq (%)	Reads	Reads (%)	ES	
IPA	2060	0.20	3747	0.21	2060	0.20	8031	0.2	2.14	1632	0.21	6909	0.21	1.84	141	0.21	253	0.19	0.07	SA1
DLR	4261	0.42	7546	0.43	4261	0.42	14205	0.4	1.88	3242	0.42	10878	0.34	1.44	290	0.43	411	0.31	0.05	
HEG	799	0.08	1393	0.08	799	0.08	2467	0.1	1.77	598	0.08	1753	0.05	1.26	42	0.06	58	0.04	0.04	
GWQ	550	0.05	1653	0.09	550	0.05	7303	0.2	4.42	438	0.06	6823	0.21	4.13	56	0.08	228	0.17	0.14	
LPS	8692	0.86	14873	0.85	8692	0.86	31233	0.8	2.10	6646	0.86	26243	0.81	1.76	602	0.90	970	0.74	0.07	
VPV	3886	0.38	7457	0.42	3886	0.38	18099	0.5	2.43	2977	0.38	14112	0.43	1.89	240	0.36	454	0.35	0.06	
AWK	488	0.05	907	0.05	488	0.05	3519	0.1	3.88	373	0.05	3768	0.12	4.15	35	0.05	119	0.09	0.13	
ERI	984	0.10	1817	0.10	984	0.10	4986	0.1	2.74	745	0.10	4131	0.13	2.27	68	0.10	125	0.10	0.07	
GLR	3949	0.39	6778	0.39	3949	0.39	14573	0.4	2.15	3181	0.41	11725	0.36	1.73	263	0.39	3709	2.84	0.55	
ANN	1507	0.15	2586	0.15	1507	0.15	4873	0.1	1.88	1391	0.18	5198	0.16	2.01	167	0.25	864	0.66	0.33	
VVP	3685	0.36	6877	0.39	3685	0.36	15934	0.4	2.32	2826	0.36	12637	0.39	1.84	251	0.38	459	0.35	0.07	
VDR	2550	0.25	4216	0.24	2550	0.25	7720	0.2	1.83	1962	0.25	5659	0.17	1.34	155	0.23	194	0.15	0.05	
MAY	751	0.07	7787	0.44	751	0.07	75242	2.0	9.66	567	0.07	89011	2.74	11.43	67	0.10	3298	2.52	0.42	
SLP	9334	0.92	15610	0.89	9334	0.92	27364	0.7	1.75	7176	0.92	20627	0.64	1.32	649	0.97	822	0.63	0.05	
TPR	3423	0.34	6439	0.37	3423	0.34	16821	0.5	2.61	2671	0.34	17254	0.53	2.68	246	0.37	670	0.51	0.10	
WPL	2126	0.21	3544	0.20	2126	0.21	6152	0.2	1.74	1774	0.23	4804	0.15	1.36	133	0.20	166	0.13	0.05	
SLR	763	0.62	793	0.62	763	0.62	908	0.61	1.15	577	0.63	977	0.36	1.23	112	0.60	264	0.06	0.33	SA2
DVP	462	0.37	474	0.37	462	0.37	530	0.36	1.12	297	0.33	398	0.15	0.84	64	0.34	103	0.02	0.22	
TGP	474	0.38	524	0.41	474	0.38	671	0.45	1.28	326	0.36	1057	0.39	2.02	64	0.34	384	0.08	0.73	
LER	286	0.23	289	0.22	286	0.23	320	0.22	1.11	218	0.24	303	0.11	1.05	51	0.27	77	0.02	0.27	
LPL	1028	0.83	1066	0.83	1028	0.83	1198	0.81	1.12	757	0.83	1259	0.46	1.18	155	0.83	313	0.07	0.29	
YWR	50	0.04	50	0.04	50	0.04	60	0.04	1.20	48	0.05	99	0.04	1.98	11	0.06	31	0.01	0.62	
TLR	578	0.47	605	0.47	578	0.47	692	0.47	1.14	437	0.48	837	0.31	1.38	91	0.49	237	0.05	0.39	
EPA	212	0.17	223	0.17	212	0.17	266	0.18	1.19	176	0.19	336	0.12	1.51	27	0.14	88	0.02	0.39	
MPI	159	0.13	168	0.13	159	0.13	210	0.14	1.25	113	0.12	284	0.10	1.69	19	0.10	105	0.02	0.63	
DLR	472	0.38	481	0.37	472	0.38	538	0.36	1.12	406	0.45	531	0.19	1.10	90	0.48	121	0.03	0.25	
THE	156	0.13	159	0.12	156	0.13	170	0.11	1.07	126	0.14	147	0.05	0.92	20	0.11	24	0.01	0.15	
PLK	260	0.21	264	0.21	260	0.21	299	0.20	1.13	224	0.25	364	0.13	1.38	38	0.20	133	0.03	0.50	
IER	111	0.09	113	0.09	111	0.09	122	0.08	1.08	107	0.12	161	0.06	1.42	23	0.12	36	0.01	0.32	
TYG	260	0.21	272	0.21	260	0.21	313	0.21	1.15	159	0.17	283	0.10	1.04	58	0.31	107	0.02	0.39	
VPV	499	0.40	504	0.39	499	0.40	567	0.38	1.13	409	0.45	600	0.22	1.19	83	0.45	139	0.03	0.28	
WPL	279	0.23	300	0.23	279	0.23	387	0.26	1.29	204	0.22	542	0.20	1.81	51	0.27	164	0.03	0.55	
RIP	282	0.23	288	0.22	282	0.23	316	0.21	1.10	186	0.20	254	0.09	0.88	48	0.26	91	0.02	0.32	

Supplementary Table A6. Overview of the number and percentage of unique sequences as well as the number and percentage of absolute reads containing the hexapeptide motifs discovered by the STREME analysis in the naive library (NL), round 1 (R1), round 2 (R2), and round 3 (R3) of both SA experiments and the ES of these motifs during amplification rounds. Each column for each SA experiment has been color-weighed by a color gradient from low value with light green to high value with dark blue.

	NL				R1					R2					R3					
Motifs	Unique seq	Unique seq (%)	Reads	Reads (%)	Unique seq	Unique seq (%)	Reads	Reads (%)	ES	Unique seq	Unique seq (%)	Reads	Reads (%)	ES	Unique seq	Unique seq (%)	Reads	Reads (%)	ES	
DLPSLR	2	0.00020	2	0.00011	2	0.00020	2	0.00005	1.00	1	0.00013	3	0.00009	1.50	0	0.00000	0	0.00000	0.00	
PVLSST	2	0.00020	2	0.00011	2	0.00020	2	0.00005	1.00	2	0.00026	2	0.00006	1.00	0	0.00000	0	0.00000	0.00	
SYTLSH	3	0.00030	70	0.00398	3	0.00030	132	0.00354	1.89	2	0.00026	224	0.00690	3.20	2	0.00300	8	0.00612	0.11	
ITGLTT	0	0.00000	0	0.00000	0	0.00000	0	0.00000	0.00	1	0.00013	1	0.00003	0.00	2	0.00300	2	0.00153	0.00	
GRAMAY	16	0.00158	6514	0.37019	16	0.00158	72592	1.94901	11.14	16	0.00206	86773	2.67275	13.32	11	0.01650	3221	2.46397	0.49	
VSVPGS	4	0.00040	6	0.00034	4	0.00040	14	0.00038	2.33	3	0.00039	10	0.00031	1.67	0	0.00000	0	0.00000	0.00	
SVSRYL	1	0.00010	4	0.00023	1	0.00010	42	0.00113	10.50	3	0.00039	39	0.00120	9.75	1	0.00150	1	0.00076	0.25	SA1
IANSTD	0	0.00000	0	0.00000	0	0.00000	0	0.00000	0.00	1	0.00013	4	0.00012	0.00	0	0.00000	0	0.00000	0.00	
RAMAYS	17	0.00168	6516	0.37031	17	0.00168	72602	1.94928	11.14	17	0.00219	86808	2.67382	13.32	13	0.01949	3223	2.46550	0.49	
GIAHSV	1	0.00010	1	0.00006	1	0.00010	5	0.00013	5.00	1	0.00013	4	0.00012	4.00	0	0.00000	0	0.00000	0.00	
NSPSLT	1	0.00010	1	0.00006	1	0.00010	4	0.00011	4.00	1	0.00013	1	0.00003	1.00	1	0.00150	1	0.00076	1.00	
DSTRGV	1	0.00010	3	0.00017	1	0.00010	3	0.00008	1.00	1	0.00013	1	0.00003	0.33	1	0.00150	1	0.00076	0.33	
MAYSTI	16	0.00158	6511	0.37002	16	0.00158	72597	1.94914	11.15	16	0.00206	86802	2.67364	13.33	12	0.01800	3222	2.46473	0.49	
DTLLRS	0	0.00000	0	0.00000	0	0.00000	0	0.00000	0.00	0	0.00000	0	0.00000	0.00	0	0.00000	0	0.00000	0.00	
AGMPCL	0	0.00000	0	0.00000	0	0.00000	0	0.00000	0.00	0	0.00000	0	0.00000	0.00	0	0.00000	0	0.00000	0.00	
HSPRPA	0	0.00000	0	0.00000	0	0.00000	0	0.00000	0.00	0	0.00000	0	0.00000	0.00	0	0.00000	0	0.00000	0.00	
NNPRSR	1	0.00081	1	0.00078	1	0.00081	1	0.00068	1.00	1	0.00110	1	0.00037	1.00	0	0.00000	0	0.00000	0.00	
DSSGLR	2	0.00162	4	0.00311	2	0.00162	5	0.00338	1.25	2	0.00219	7	0.00256	1.75	1	0.00537	1	0.00021	0.25	
NAITTR	1	0.00081	1	0.00078	1	0.00081	2	0.00135	2.00	1	0.00110	1	0.00037	1.00	0	0.00000	0	0.00000	0.00	
DPNRLS	1	0.00081	1	0.00078	1	0.00081	2	0.00135	2.00	1	0.00110	7	0.00256	7.00	1	0.00537	7	0.00149	7.00	SA2
TIRDLP	0	0.00000	0	0.00000	0	0.00000	0	0.00000	0.00	1	0.00110	1	0.00037	0.00	0	0.00000	0	0.00000	0.00	
SSPLGF	1	0.00081	1	0.00078	1	0.00081	2	0.00135	2.00	1	0.00110	3	0.00110	3.00	0	0.00000	0	0.00000	0.00	
SPSLAL	0	0.00000	0	0.00000	0	0.00000	0	0.00000	0.00	2	0.00219	2	0.00073	0.00	0	0.00000	0	0.00000	0.00	
STYGPV	0	0.00000	0	0.00000	0	0.00000	0	0.00000	0.00	0	0.00000	0	0.00000	0.00	1	0.00537	1	0.00021	0.00	
HLTLQT	1	0.00081	1	0.00078	1	0.00081	1	0.00068	1.00	0	0.00000	0	0.00000	0.00	1	0.00537	1	0.00021	1.00	
WPLSWN	0	0.00000	0	0.00000	0	0.00000	0	0.00000	0.00	0	0.00000	0	0.00000	0.00	1	0.00537	1	0.00021	0.00	
AVTDFL	0	0.00000	0	0.00000	0	0.00000	0	0.00000	0.00	0	0.00000	0	0.00000	0.00	1	0.00537	1	0.00021	0.00	

Supplementary Table A7. Overview of the number and percentage of unique sequences, the number and percentage of absolute reads and the number of N-terminal placements (sequences that contain the motif at their N-termini) of the eight tetramer motifs associated with β-turn formation in the secondary structure of displayed peptides in the naive library (NL), round 1 (R1), round 2 (R2), and round 3 (R3) of both experiments and the ES of these motifs during amplification rounds. Each column for each SA experiment has been color-weighed by a color gradient from low value with light green to high value with dark blue.

	NL					R1						
Motifs	Unique seq	Unique seq (%)	Reads	Reads (%)	N-terminus	Unique seq	Unique seq (%)	Reads	Reads (%)	N-terminus	ES	
PPNP	130	0.01284	237	0.01347	0	130	0.01284	343	0.00921	0	1.45	SA1
DPGG	126	0.01245	208	0.01182	13	126	0.01245	283	0.00760	13	1.36	SA1
YGNT	98	0.00968	145	0.00824	17	98	0.00968	206	0.00553	17	1.42	SA1
DPNG	76	0.00751	100	0.00568	10	76	0.00751	199	0.00534	10	1.99	SA1
DPDG	72	0.00711	95	0.00540	13	72	0.00711	138	0.00371	13	1.45	SA1
PNWN	43	0.00425	79	0.00449	0	43	0.00425	111	0.00298	0	1.41	SA1
PPGC	40	0.00395	68	0.00386	0	40	0.00395	87	0.00234	0	1.28	SA1
YNGK	24	0.00237	35	0.00199	7	24	0.00237	51	0.00137	7	1.46	SA1
PPNP	23	0.01861	23	0.01790	0	23	0.01861	26	0.01757	0	1.13	SA2
DPGG	21	0.01699	21	0.01634	3	21	0.01699	24	0.01622	3	1.14	SA2
DPDG	13	0.01052	13	0.01012	0	13	0.01052	13	0.00879	0	1.00	SA2
DPNG	13	0.01052	13	0.01012	1	13	0.01052	14	0.00946	1	1.08	SA2
YGNT	7	0.00566	7	0.00545	2	7	0.00566	7	0.00473	2	1.00	SA2
PNWN	6	0.00485	6	0.00467	0	6	0.00485	8	0.00541	0	1.33	SA2
PPGC	6	0.00485	6	0.00467	0	6	0.00485	7	0.00473	0	1.17	SA2
YNGK	1	0.00081	1	0.00078	0	1	0.00081	1	0.00068	0	1.00	SA2

	R2						R3						
Motifs	Unique seq	Unique seq (%)	Reads	Reads (%)	N-terminus	ES	Unique seq	Unique seq (%)	Reads	Reads (%)	N-terminus	ES	
PPNP	115	0.0148	274	0.00844	0	1.16	12	0.0180	12	0.00918	0	0.051	SA1
DPGG	90	0.0116	185	0.00570	10	0.89	7	0.0105	7	0.00535	0	0.034	SA1
YGNT	61	0.0079	133	0.00410	13	0.92	3	0.0045	3	0.00229	0	0.021	SA1
DPNG	60	0.0077	158	0.00487	6	1.58	9	0.0135	9	0.00688	1	0.090	SA1
DPDG	55	0.0071	99	0.00305	11	1.04	5	0.0075	5	0.00382	0	0.053	SA1
PNWN	32	0.0041	81	0.00249	0	1.03	3	0.0045	3	0.00229	0	0.038	SA1
PPGC	13	0.0017	22	0.00068	0	0.32	1	0.0015	1	0.00076	0	0.015	SA1
YNGK	22	0.0028	36	0.00111	4	1.03	0	0.0000	0	0	0	0	SA1
PPNP	14	0.0154	17	0.00623	0	0.74	2	0.0107	2	0.00042	0	0.087	SA2
DPGG	9	0.0099	9	0.00330	0	0.43	2	0.0107	2	0.00042	0	0.095	SA2
DPDG	11	0.0121	11	0.00403	2	0.85	2	0.0107	2	0.00042	0	0.154	SA2
DPNG	10	0.0110	20	0.00733	0	1.54	2	0.0107	2	0.00042	0	0.154	SA2
YGNT	6	0.0066	6	0.00220	4	0.86	1	0.0054	1	0.00021	0	0.143	SA2
PNWN	6	0.0066	15	0.00549	0	2.50	2	0.0107	7	0.00149	0	1.167	SA2
PPGC	2	0.0022	2	0.00073	0	0.33	0	0.0000	0	0	0	0	SA2
YNGK	0	0.0000	0	0.00000	0	0	0	0.0000	0	0	0	0	SA2
